# Cumulative Frameworks as a Pragmatic Alternative to Multivariable Modeling in Rare-Event Clinical Settings: A Coronary Care Unit Case Study

**DOI:** 10.3390/mps9030092

**Published:** 2026-06-10

**Authors:** Daniela Mirela Vîrtosu, Simina Crișan, Oana Pătru, Angela Dragomir, Silvia Luca, Ruxandra-Maria Băghină, Mihai-Andrei Lazăr, Alina-Ramona Cozlac, Stela Iurciuc, Constantin Tudor Luca

**Affiliations:** 1 Doctoral School, “Victor Babes” University of Medicine and Pharmacy, 300041 Timisoara, Romania; daniela.cozma@umft.ro (D.M.V.); oana.patru@umft.ro (O.P.); silvia.luca@umft.ro (S.L.); ruxandra.croicu@umft.ro (R.-M.B.); 2 Institute of Cardiovascular Diseases Timisoara, 13A Gheorghe Adam Street, 300310 Timisoara, Romania; angela.dragomir@umft.ro (A.D.); lazar.mihai@umft.ro (M.-A.L.); alina-ramona.cozlac@umft.ro (A.-R.C.); constantin.luca@umft.ro (C.T.L.); 3 Research Center of the Institute of Cardiovascular Diseases Timisoara, 13A Gheorghe Adam Street, 300310 Timisoara, Romania; 4 Cardiology Department, “Victor Babes” University of Medicine and Pharmacy, 2 Eftimie Murgu Sq., 300041 Timisoara, Romania; iurciuc.stela@umft.ro

**Keywords:** rare-event modeling, events per variable, cumulative framework, coronary care unit, healthcare-associated infections, overfitting, clinical risk stratification

## Abstract

**Background**: Risk stratification models are widely used in clinical research; however, their development becomes methodologically challenging in settings characterized by low outcome incidence. In coronary care unit (CCU) populations, healthcare-associated infections (HAIs) occur relatively infrequently, limiting the feasibility of conventional multivariable predictive modeling. **Methods**: A retrospectively assembled CCU cohort comprising 870 patients with 16 HAI events (1.8%) was used as an illustrative example to examine methodological constraints associated with low events-per-variable (EPV) ratios. The implications of limited event frequency for multivariable logistic regression were evaluated, including risks of overfitting, coefficient instability, and reduced reproducibility. As an alternative strategy, a prespecified cumulative additive framework integrating baseline vulnerability and exposure-related variables was conceptually and analytically explored. **Results**: With four candidate predictors and 16 outcome events, the resulting EPV was approximately four, indicating a high risk of instability for conventional multivariable modeling. The cumulative framework allowed structured cumulative stratification without coefficient optimization. Infection occurrence increased progressively across cumulative framework levels, illustrating a graded pattern of increasing HAI occurrence with accumulating vulnerability and exposure-related burden. **Conclusions**: In clinical datasets with limited outcome events, modeling strategies should be aligned with the informational capacity of the data. Cumulative additive frameworks may represent a pragmatic structural alternative to coefficient-based modeling approaches in rare-event clinical settings. The present work provides a structured methodological framework for risk stratification under low-events-per-variable conditions rather than proposing a novel clinical scoring system.

## 1. Introduction

Risk stratification models play a central role in contemporary clinical research and clinical decision-making [1]. In critical care settings, predictive frameworks are frequently developed to identify patients at increased risk of adverse outcomes, guide preventive strategies, and optimize resource allocation. Multivariable logistic regression remains the most widely applied analytical approach for estimating associations between candidate predictors and clinical outcomes [1,2]. However, the reliability of regression-based models depends heavily on the relationship between the number of outcome events and the number of predictors included in the model.

In coronary care unit (CCU) populations, healthcare-associated infections (HAIs) represent clinically relevant complications, yet their incidence is typically lower than that observed in general intensive care settings [3]. Although this relatively low event frequency reflects effective infection-prevention practices, it simultaneously introduces methodological constraints for predictive model development. When outcome events are limited, conventional multivariable regression approaches become vulnerable to overfitting, coefficient instability, inflated variance, and reduced reproducibility [4]. The widely discussed concept of events per variable (EPV) highlights the risk of unstable parameter estimation when the number of candidate predictors approaches or exceeds the available number of outcome events. Classical recommendations have suggested that approximately ten outcome events per predictor variable may be required to ensure stable coefficient estimation, although subsequent methodological research has shown that model performance depends on multiple factors including event frequency, predictor distribution, and overall model complexity [5,6,7].

Previous analyses of CCU cohorts have highlighted the multifactorial nature of infection risk, reflecting the interaction between baseline vulnerability and cumulative exposure to invasive care [8,9]. Rare-event contexts therefore create a fundamental methodological tension between the increasing demand for refined and individualized risk prediction and the statistical constraints imposed by limited information content. In small-event cohorts, aggressive variable selection procedures, data-driven cut-off optimization, or extensive model tuning may yield apparently significant associations that reflect sampling variability rather than reproducible relationships [10]. These challenges are particularly relevant in highly specialized clinical environments such as CCUs, where patient populations may be relatively homogeneous and outcome events inherently infrequent.

Under such conditions, alternative approaches to risk stratification may warrant consideration. Rather than pursuing coefficient-weighted multivariable prediction models, prespecified cumulative additive frameworks may offer advantages in transparency, interpretability, and structural stability. Simple additive models, when grounded in biological plausibility and clinical reasoning, may reduce the risk of overfitting while preserving clinically meaningful stratification [1]. Nevertheless, cumulative frameworks also raise important methodological questions, including the selection of thresholds for dichotomization, the handling of time-dependent exposures, and the potential introduction of bias when cumulative variables such as length of stay (LOS) are incorporated into the model structure.

The objective of the present study is not to introduce a novel predictive algorithm, but rather to examine methodological challenges that arise when cumulative risk frameworks are applied in datasets with limited outcome events. Using a CCU cohort with a low incidence of HAIs as an illustrative case example, this study explores the constraints associated with conventional multivariable modeling and discusses the rationale for adopting a cumulative additive framework in rare-event settings. By doing so, we aim to provide practical methodological guidance for selecting risk stratification strategies that are proportionate to the informational capacity of the available data. The proposed contribution therefore lies not in the development of a new clinical scoring system, but in the formulation of a structured methodological framework that prioritizes interpretability, structural stability, and transparency when event frequency limits the feasibility of conventional predictive modeling.

## 2. Methods

### 2.1. Study Context and Illustrative Dataset

To explore methodological challenges in rare-event risk modeling, a retrospectively assembled CCU cohort was used as an illustrative case example. The cohort was assembled by the study investigators at the Institute of Cardiovascular Diseases Timisoara and comprised 870 consecutive adult patients admitted during a predefined six-month study period, among whom 16 HAIs were documented, corresponding to an incidence of 1.8%. This cohort has been described previously in a clinical study investigating risk factors for healthcare-associated infections in CCU patients and is used here solely as an illustrative example for methodological purposes [9].

The dataset was incorporated solely to illustrate the methodological constraints encountered when outcome events are limited relative to the number of candidate predictors. The objective of the present analysis was not to perform additional predictive modeling or to develop or validate a clinical prediction framework, but rather to demonstrate how limited event frequency can restrict the feasibility and stability of conventional multivariable regression approaches.

The illustrative dataset included clinically relevant variables representing both baseline patient vulnerability and exposure-related factors commonly considered in infection risk assessment within cardiac critical care environments.

No new data were collected for the purposes of this methodological analysis. All evaluations were performed using anonymized data previously collected by the study investigators as part of routine clinical care and subsequently analyzed in the previously published cohort study.

### 2.2. Assessment of Modeling Feasibility Under Limited Event Conditions

The primary methodological consideration was the relationship between the number of observed outcome events and the number of candidate predictors intended for inclusion in a multivariable model. This relationship is commonly summarized using the EPV ratio, calculated as the number of outcome events divided by the number of predictors included in the model.

In the illustrative cohort, 16 HAI events were observed. Four clinically relevant candidate predictors were considered: reduced left ventricular ejection fraction (LVEF < 40%), diabetes mellitus, urinary catheterization, and prolonged CCU length of stay (>5 days). These variables were selected a priori based on biological plausibility and clinical relevance within the CCU setting. Considering these four candidate predictors, the resulting EPV was approximately four. This value falls substantially below commonly cited thresholds historically considered necessary for stable coefficient estimation in logistic regression models.

The methodological implications of this constraint were evaluated conceptually, focusing on potential risks associated with low EPV values, including model overfitting, unstable coefficient estimates, inflated variance, and limited reproducibility. Rather than applying penalized regression or rare-event correction techniques, the analysis focused on examining alternative structural modeling strategies that prioritize interpretability and stability when event counts are inherently limited.

### 2.3. Conceptualization of the Cumulative Additive Framework

As an alternative to coefficient-weighted multivariable modeling, a cumulative additive framework was conceptually constructed. Candidate variables were prespecified based on biological plausibility and clinical relevance within the CCU setting rather than selected through data-driven optimization procedures. The framework incorporated four variables: reduced left ventricular ejection fraction (LVEF) (LVEF < 40%), diabetes mellitus, urinary catheterization, and prolonged CCU length of stay (>5 days). These variables were selected to represent two complementary domains commonly implicated in HAI development: baseline patient vulnerability (reduced LVEF and diabetes mellitus) and exposure-related risk accumulated during hospitalization (urinary catheterization and prolonged length of stay).

Each variable was dichotomized using clinically meaningful thresholds and assigned one point when present. Reduced LVEF was defined as <40%, consistent with established definitions of systolic dysfunction, while prolonged CCU stay was defined as >5 days based on clinical reasoning and cohort distribution characteristics. Equal weighting (1 point per factor) was intentionally adopted to prioritize simplicity, transparency, and bedside applicability rather than statistical optimization. The cumulative framework therefore reflected the progressive accumulation of vulnerability and exposure-related burden across individual patients.

Importantly, threshold selection was determined a priori rather than through receiver-operating-characteristic optimization or post hoc cut-off derivation. This pre-specification was intended to reduce the risk of data-driven overfitting and to enhance structural transparency and reproducibility.

### 2.4. Analytical Strategy

Descriptive analyses were performed to illustrate the distribution of cumulative frameworks and the corresponding occurrence of outcome events across framework categories. In the original cohort study from which the illustrative example was derived, exploratory logistic regression analysis demonstrated a monotonic association between increasing cumulative framework levels and HAI occurrence (OR 2.48, 95% CI 1.61–3.82; *p* < 0.001). The present manuscript does not seek to re-estimate these effects, but rather uses the previously reported findings to illustrate the methodological concepts discussed herein [9].

Importantly, this analysis was conducted solely for illustrative purposes and was not intended to derive optimized predictive coefficients or to construct a validated prediction model. No multivariable coefficient optimization, internal validation procedures, shrinkage techniques, or discrimination metrics (such as area under the receiver operating characteristic curve) were pursued.

The analytical approach was therefore intentionally restricted to demonstrating the structural behavior of a cumulative additive framework under limited event conditions rather than maximizing predictive performance.

### 2.5. Implementation Protocol for Risk Stratification in the CCU Setting

To translate the conceptual framework into a clinically applicable approach, a pragmatic implementation protocol was defined for use in the CCU setting. The proposed pathway illustrates how the cumulative framework may be applied in routine clinical practice and is intended as a methodological example rather than a validated clinical protocol.

#### 2.5.1. Target Population and Timing of Assessment

The protocol is intended for application to all adult patients admitted to the CCU. Baseline vulnerability factors are assessed at admission, while exposure-related variables are evaluated dynamically throughout hospitalization. Risk stratification is therefore conceived as an evolving process reflecting cumulative burden over time.

#### 2.5.2. Selection and Definition of Variables

Variables included in the cumulative framework are prespecified based on clinical plausibility and relevance to infection risk in critical care. Detailed justification and threshold definitions for individual variables are provided in Section 2.3. Within the implementation protocol, variables are categorized as baseline vulnerability factors (assessed at admission) or exposure-related factors (reassessed during hospitalization).

All variables are operationalized as dichotomous indicators to facilitate consistent application and reduce model complexity.

#### 2.5.3. Stepwise Implementation Procedure

The protocol follows a structured sequence of steps:

Step 1: Assess baseline vulnerability at admission, including LVEF and diabetes status.

Step 2: Monitor exposure-related variables during hospitalization, including urinary catheterization and duration of CCU stay.

Step 3: Assign one point for each variable present.

Step 4: Calculate the cumulative framework as the unweighted sum of all present factors, resulting in a cumulative value ranging from 0 to 4.

Step 5: Stratify patients according to cumulative burden, with higher framework values reflecting progressively greater accumulation of vulnerability and exposure-related factors.

#### 2.5.4. Dynamic Updating of Cumulative Framework

Because exposure-related variables evolve over time, the cumulative framework should be updated periodically during hospitalization. This dynamic approach allows cumulative stratification to reflect the progressive accumulation of vulnerability and exposure, rather than relying solely on baseline assessment.

Baseline vulnerability factors, including LVEF and diabetes mellitus, are assessed at admission and remain unchanged throughout hospitalization. Exposure-related variables, including urinary catheterization and CCU LOS, are reassessed daily as part of routine clinical care. The cumulative score is recalculated whenever a new exposure-related criterion becomes present, allowing risk stratification to evolve in parallel with the patient’s clinical course.

#### 2.5.5. Clinical Interpretation and Use

The cumulative framework is intended to support pragmatic cumulative stratification rather than precise individualized prediction. Higher framework values may help identify patients with greater accumulation of vulnerability and exposure-related factors, potentially supporting closer infection-prevention awareness and clinical surveillance.

Importantly, the framework is primarily intended as a bedside risk-stratification tool that can be implemented within routine clinical workflows. The protocol prioritizes transparency and ease of implementation, allowing real-time risk assessment without reliance on coefficient-weighted modeling, electronic decision-support systems, or advanced computational resources. All framework components are directly observable in routine clinical practice, facilitating practical application in settings where event frequency is limited and model interpretability is particularly important.

#### 2.5.6. Scope and Limitations of the Protocol

This protocol is not intended to replace validated predictive models or to provide precise probability estimates of infection risk. Instead, it offers a structured approach to risk stratification in settings where limited outcome events constrain the feasibility of conventional multivariable modeling.

The framework should therefore be interpreted as a pragmatic tool aligned with the informational capacity of rare-event clinical datasets, and its application should be accompanied by awareness of potential temporal and exposure-related biases discussed in the methodological sections above.

**Figure 1:**
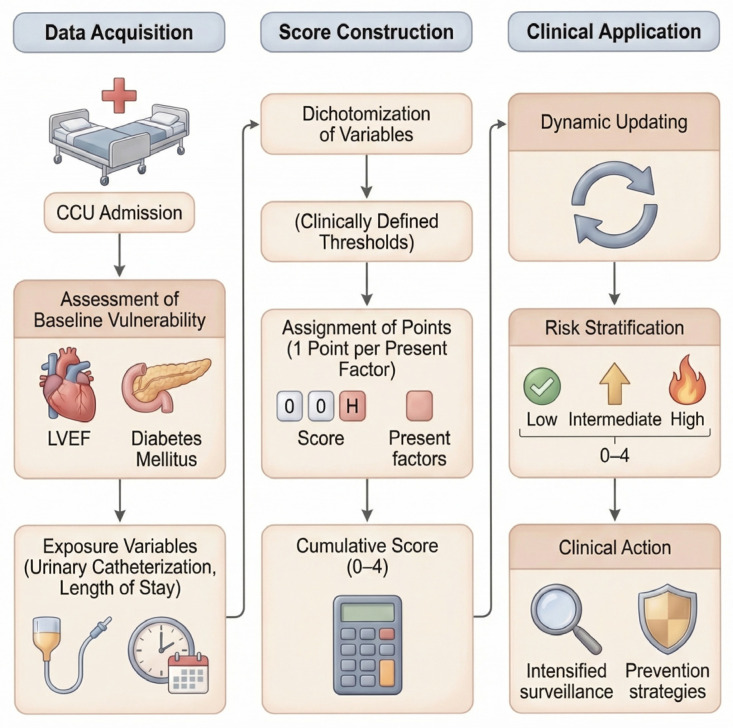
Structured methodological framework for risk stratification in rare-event clinical settings. The diagram defines a stepwise approach to cumulative risk assessment in CCU patients. Baseline vulnerability factors (e.g., LVEF, diabetes mellitus) are assessed at admission, while exposure-related variables (e.g., urinary catheterization, LOS) are monitored during hospitalization. Variables are dichotomized using clinically defined thresholds and assigned equal weight within a cumulative additive score (0–4). The score is dynamically updated to reflect evolving patient status and used to stratify patients into risk categories, supporting targeted infection prevention and surveillance strategies.

The structured methodological framework and its implementation in the coronary care unit setting are summarized in Figure 1.

## 3. The Challenge of Limited Event Counts in Clinical Risk Modeling

### 3.1. Events per Variable and Model Stability

Multivariable logistic regression remains the dominant framework for clinical risk modeling [11]. However, the stability and reliability of regression-based models depend critically on the relationship between the number of outcome events and the number of candidate predictors included in the model. The concept of EPV has long been used as a heuristic indicator of modeling feasibility.

Early methodological studies suggested that approximately ten outcome events per predictor variable may be required to ensure stable coefficient estimation and acceptable variance in logistic regression models [5]. Subsequent methodological research has shown that this rule should not be interpreted as a strict threshold, as model stability also depends on factors such as predictor distribution, effect size, and overall model complexity [6,7]. Nevertheless, low EPV values remain associated with an increased risk of unstable parameter estimation and overfitting [4].

When EPV falls substantially below commonly recommended ranges, several statistical consequences may emerge. Regression coefficients may become unstable, standard errors inflate, and confidence intervals widen disproportionately. Variable selection procedures, particularly stepwise approaches, may further amplify instability, producing models that appear statistically significant within the derivation dataset but fail to replicate in independent samples.

In rare-event contexts such as the present CCU example, where 16 outcome events were observed, inclusion of four candidate predictors yields an EPV of approximately four. Under such conditions, regression coefficients may become highly sensitive to minor perturbations in the dataset, increasing the likelihood that estimated effects reflect sampling variability rather than reproducible signal. Apparent statistical significance in such models does not necessarily imply structural reliability.

Under the traditional EPV ≈ 10 heuristic, a model including four predictors would require approximately 40 outcome events to achieve the recommended stability range.

### 3.2. Overfitting and Optimism in Small-Event Models

Overfitting occurs when a statistical model captures random variation specific to the derivation dataset rather than underlying population structure. In low-event settings, the risk of overfitting increases substantially, particularly when multiple predictors, interaction terms, or data-driven threshold optimizations are employed [4].

A common manifestation of overfitting is optimism in model performance. Discrimination metrics such as the area under the receiver operating characteristic curve (AUC) may appear favorable in the derivation dataset but deteriorate when the model is evaluated in independent cohorts [11]. Internal validation procedures, including bootstrapping or cross-validation, may help quantify optimism but cannot fully compensate for the limited information content inherent to small-event datasets.

Importantly, the pursuit of maximal statistical discrimination through increasingly complex model structures may paradoxically reduce generalizability when event counts are limited. In such situations, additional model flexibility may increase apparent performance while simultaneously amplifying instability and reducing reproducibility.

### 3.3. Rare-Event Regression and Penalization: Solutions and Limitations

Several alternative modeling strategies have been proposed to address small-event contexts, including penalized regression techniques such as ridge regression or least absolute shrinkage and selection operator (LASSO), bias-reduced logistic regression approaches such as Firth’s method, and Bayesian frameworks incorporating informative priors. These approaches aim to mitigate small-sample bias, shrink unstable coefficients, and improve parameter estimation in datasets with limited outcome events [12,13,14].

While methodologically appealing, these techniques introduce additional analytical complexity. Penalized regression approaches require the selection of tuning parameters, which may themselves be unstable in limited datasets. Rare-event corrections can reduce bias in coefficient estimation but do not fundamentally increase the information content available for model construction. Furthermore, increased algorithmic complexity may reduce transparency and hinder clinical interpretability.

In specialized clinical environments such as the CCU, where the primary objective may be pragmatic risk stratification rather than precise individualized probability estimation, the incremental benefit of advanced modeling techniques must therefore be carefully balanced against considerations of interpretability and reproducibility. Accordingly, advanced rare-event modeling approaches and cumulative additive frameworks should be viewed as complementary rather than competing strategies, with their selection guided by the intended clinical application and the informational capacity of the available data.

Future methodological investigations using larger datasets and greater numbers of outcome events may help clarify the relative performance of cumulative additive frameworks, conventional regression models, bias-reduced approaches, and penalized regression techniques. Such comparisons could provide further insight into the trade-offs between predictive performance, coefficient stability, susceptibility to overfitting, reproducibility, and clinical interpretability across different rare-event settings.

### 3.4. Structural Simplicity as a Methodological Choice

In rare-event settings, methodological restraint may represent a defensible alternative to statistical sophistication. When outcome frequency constrains model stability, structurally simple frameworks based on prespecified variables may provide greater robustness than highly optimized multivariable prediction models [1,11].

Rather than maximizing coefficient precision, cumulative additive approaches prioritize transparency and structural consistency. By avoiding data-driven variable weighting, stepwise selection procedures, and threshold optimization, such frameworks reduce the risk of fitting random noise inherent to small-event datasets.

This trade-off reflects a broader methodological principle: modeling strategy should align with the informational capacity of the available data. In contexts where the number of outcome events is inherently limited, prioritizing interpretability, transparency, and structural stability may represent a rational and defensible methodological choice.

### 3.5. When May a Cumulative Additive Framework Be Considered?

The cumulative additive framework proposed in this manuscript is not intended as a universal replacement for conventional regression-based prediction models. Rather, it may be particularly relevant in situations where the informational capacity of the available data limits the feasibility or stability of highly parameterized approaches.

Examples include datasets characterized by low EPV ratios, small numbers of outcome events, limited sample sizes, or circumstances in which coefficient estimates are expected to be unstable or poorly reproducible [4,5,6,7]. In such settings, simpler cumulative approaches may offer advantages in terms of interpretability, transparency, implementation feasibility, and structural robustness.

Frequently cited methodological indicators, such as EPV values below approximately five or very small numbers of outcome events, should not be interpreted as absolute decision thresholds. Instead, they may serve as practical signals prompting investigators to carefully evaluate whether model complexity remains proportionate to the information available within the dataset.

Ultimately, the selection of a cumulative additive framework versus more advanced modeling techniques should be guided by the study objective, data structure, event frequency, and intended clinical application. The framework may be particularly useful when the primary goal is pragmatic risk stratification and clinical interpretability rather than precise individualized probability estimation.

## 4. Case Example: Application in a CCU Cohort

### 4.1. Cohort Characteristics and Event Distribution

The illustrative cohort consisted of 870 consecutive patients admitted to a CCU, among whom 16 HAIs were documented, corresponding to an overall incidence of 1.8%. This relatively low event frequency reflects the epidemiological reality of infection occurrence in specialized cardiac critical care environments, where infection-prevention practices are routinely implemented and patient populations may be more homogeneous than in general ICUs.

Four clinically plausible candidate predictors were considered for infection risk stratification: LVEF, diabetes mellitus, urinary catheterization during hospitalization, and prolonged CCU LOS. These variables were selected based on biological plausibility and previously reported associations with infection susceptibility in critically ill populations [9].

With 16 outcome events and four candidate predictors, the resulting EPV ratio was approximately four. This value falls below commonly suggested stability ranges for multivariable regression models and illustrates the methodological constraints associated with attempting coefficient-weighted predictive modeling in small-event datasets.

### 4.2. Hypothetical Multivariable Modeling Constraints

If a conventional multivariable logistic regression model had been constructed incorporating these four predictors simultaneously, several methodological concerns would arise. First, coefficient estimates would likely exhibit substantial variance and sensitivity to minor perturbations in the dataset. Second, standard errors would be inflated, resulting in reduced precision and wider confidence intervals. Third, model performance estimates derived from the same dataset would be vulnerable to optimism bias.

Additional analytical procedures frequently used in predictive modeling, such as interaction testing, nonlinear functional forms, or data-driven threshold optimization, would further reduce the effective information available per parameter, thereby exacerbating model instability.

These considerations illustrate the tension between statistical ambition and data limitations. Although multivariable prediction models are conceptually attractive, their application must remain proportional to the informational capacity of the dataset.

### 4.3. Adoption of a Cumulative Additive Framework

In light of these constraints, a prespecified cumulative additive framework was considered as an alternative modeling strategy. Instead of assigning coefficient-based weights to individual predictors, each clinically relevant factor contributed one point to a cumulative burden score.

This additive structure eliminated the need for coefficient optimization and reduced susceptibility to overfitting. The resulting score therefore represented the progressive accumulation of vulnerability and exposure-related factors across patients.

Logistic regression analysis was subsequently performed with the cumulative score entered as an ordinal predictor, not to derive optimized coefficients but to illustrate the presence of a monotonic pattern of increasing infection occurrence.

This illustrative example demonstrates how modeling strategy may be adapted to align with event-frequency constraints. Rather than prioritizing maximal predictive discrimination, the cumulative approach emphasizes structural transparency and robustness in low-event contexts.

### 4.4. Illustrative Distribution of Cumulative Scores and Infection Events

To demonstrate the structural behavior of the cumulative framework, the distribution of patients and infection events across cumulative score categories was examined.

A progressive increase in infection incidence was observed with increasing cumulative burden. No infections occurred among patients with a score of 0, whereas higher score categories were associated with progressively greater infection frequency. Although the number of outcome events remained limited, this distribution illustrates the monotonic relationship between cumulative vulnerability and infection occurrence.

The distribution of infection events across cumulative score categories is summarized in Table 1.

Despite the limited number of events, the cumulative distribution suggests a consistent stepwise gradient in infection risk with increasing score levels. This pattern illustrates the potential utility of cumulative burden frameworks for pragmatic risk stratification in datasets where conventional multivariable modeling may be statistically unstable.

## 5. Bias and Temporal Considerations in Exposure-Based Risk Models

### 5.1. Reverse Causality and LOS Circularity

In cumulative risk frameworks that incorporate exposure-related variables, temporal relationships require careful scrutiny. LOS, for example, is widely recognized as a determinant of HAI risk, reflecting increased environmental exposure, device utilization, and cumulative procedural burden. However, infection itself may prolong hospitalization, introducing potential bidirectionality.

This dual relationship raises the possibility of reverse causality: prolonged LOS may increase infection risk, but infection may also extend LOS. In retrospective datasets lacking detailed time-dependent modeling, disentangling these temporal sequences is challenging. Consequently, LOS-based thresholds should be interpreted primarily as markers of cumulative exposure rather than strictly causal predictors [15].

In the present illustrative cohort, LOS was therefore conceptualized as a proxy for sustained exposure to the hospital environment rather than as an independent etiologic determinant of infection. Nevertheless, the possibility of partial circularity cannot be excluded. Explicit acknowledgment of this temporal ambiguity is important when interpreting cumulative exposure frameworks in observational datasets.

### 5.2. Time-Dependent Bias in Exposure Accumulation

Many exposure variables relevant to infection risk, including device utilization and hospitalization duration, evolve dynamically during hospitalization. When such exposures are treated as fixed dichotomous variables rather than time-dependent covariates, temporal granularity is reduced and bias may be introduced.

Time-dependent bias arises when exposure status changes during follow-up but is modeled as static. In infection research, the timing of device insertion, duration of use, and removal may substantially influence the accumulation of infection risk. Failure to account for these temporal dynamics may either overestimate or underestimate associations depending on the relative timing of exposure and outcome occurrence [16].

Survival-based approaches incorporating time-dependent covariates can address these complexities, but such models require sufficient numbers of outcome events and detailed temporal data. In small-event cohorts, highly parameterized time-dependent models may introduce additional instability. Consequently, investigators must balance temporal precision with statistical feasibility when selecting modeling strategies [16].

### 5.3. Immortal Time and Exposure Classification

Immortal time bias represents another potential concern in exposure-based observational analyses [17]. Immortal time refers to a period during which the outcome cannot occur by definition of the exposure classification. For example, if prolonged hospitalization is defined as exceeding a predetermined duration threshold, patients must survive and remain event-free long enough to meet that criterion.

If exposure classification does not properly account for this temporal structure, the resulting analysis may artificially inflate or attenuate associations between exposure and outcome. Awareness of immortal time principles is therefore essential when interpreting exposure thresholds derived from retrospective datasets.

Although the cumulative framework presented here was not designed as a survival model, careful interpretation of exposure-based variables remains important. Explicit description of exposure definitions and temporal sequencing helps reduce the risk of interpretive bias.

### 5.4. Dichotomization: Trade-Off Between Information and Interpretability

Dichotomization of continuous variables, such as LOS or LVEF, is frequently criticized for information loss and potential reductions in statistical power [18,19]. Continuous modeling approaches generally preserve more information and allow more flexible functional relationships.

However, in datasets with limited outcome events, continuous modeling may introduce additional instability and require assumptions regarding functional forms that exceed the informational capacity of the data. In such contexts, dichotomous thresholds based on clinical reasoning may represent a pragmatic compromise.

Within cumulative additive frameworks prioritizing interpretability and structural transparency, prespecified dichotomous thresholds may enhance reproducibility and facilitate bedside usability. The methodological trade-off therefore involves sacrificing some statistical granularity in order to maintain model stability and clinical interpretability. Explicit acknowledgment of this compromise helps prevent overinterpretation of simplified scoring systems.

### 5.5. Practical Strategies for Bias Mitigation

Although these methodological challenges cannot be fully eliminated in retrospective rare-event datasets, several practical measures may reduce their impact. Predictor variables should be defined and recorded before outcome occurrence whenever possible, exposure-related factors should be reassessed according to predefined assessment intervals, and temporal relationships between exposures and infection events should be explicitly documented. In addition, investigators should distinguish baseline vulnerability factors from dynamic exposure-related variables and interpret exposure-based associations primarily as markers of cumulative risk rather than definitive causal determinants. When sufficient outcome events and temporal data are available, prospective study designs and time-dependent analytical approaches may further improve the validity of cumulative risk assessment frameworks.

## 6. Interpretability Versus Predictive Performance in Rare-Event Contexts

### 6.1. The Pursuit of Discrimination

Contemporary clinical risk modeling frequently emphasizes predictive discrimination, most commonly quantified using metrics such as the AUC. High discrimination is often interpreted as evidence of model quality, and incremental gains in AUC are frequently pursued through increasingly complex modeling strategies [1,11].

However, in low-event settings, discrimination metrics may become unstable and highly sensitive to minor variations in data structure. When the number of outcome events is limited, small changes in case distribution may disproportionately influence performance estimates. Consequently, apparent improvements in discrimination may reflect sampling variability rather than meaningful predictive enhancement.

Moreover, discrimination alone does not guarantee clinical usefulness. A model may achieve acceptable AUC values while remaining poorly calibrated, difficult to interpret, or impractical for bedside application [20].

### 6.2. Transparency and Clinical Usability

In specialized clinical environments such as CCUs, interpretability and usability are essential characteristics of risk stratification tools [11]. Clinicians must understand how risk is constructed, which variables contribute to stratification, and how scores may evolve during hospitalization.

Highly parameterized models, penalized regression frameworks, or algorithmically optimized predictors may offer statistical advantages under ideal data conditions. However, these approaches frequently reduce transparency and may limit clinical uptake when implementation requires complex calculations or automated computational tools [21].

Additive cumulative frameworks prioritize structural clarity. Each component contributes equally and visibly to the overall burden score, facilitating intuitive understanding and communication within multidisciplinary teams. This transparency also supports integration into routine workflows without requiring advanced computational infrastructure.

In rare-event contexts, where predictive precision is inherently constrained by limited information, the marginal gains obtained from coefficient weighting may not justify reduced interpretability [11].

### 6.3. Stability as a Methodological Priority

Model stability, the extent to which conclusions remain consistent under minor perturbations in the dataset, is a critical but often underemphasized property of clinical prediction models. In small-event cohorts, stability may be more valuable than maximal discrimination [21].

By prespecifying variables and avoiding data-driven optimization procedures, cumulative additive frameworks reduce the likelihood that small fluctuations in dataset composition will substantially alter the resulting risk stratification structure. Although such models may not achieve optimal discrimination metrics, they may provide more reproducible and robust categorization under conditions of limited information.

Importantly, this perspective does not reject advanced modeling techniques. Instead, it emphasizes that modeling strategy should be aligned with the informational capacity of the available data. When outcome events are limited, prioritizing structural stability may represent a more defensible methodological choice.

### 6.4. Aligning Modeling Strategy with Clinical Objectives

The purpose of risk modeling in cardiac critical care is not solely to maximize statistical performance but also to support preventive strategies and clinical decision-making [11]. In low-incidence environments, identifying patients with progressively accumulating vulnerability may be more clinically relevant than estimating precise individualized outcome probabilities [7,11].

Cumulative frameworks emphasize structured risk awareness rather than deterministic prediction. They allow clinicians to recognize the progressive accumulation of vulnerability and exposure factors during hospitalization and may facilitate targeted infection-prevention strategies.

Within this context, interpretability, transparency, and reproducibility become methodological strengths rather than compromises. Balancing predictive ambition with data reality therefore represents a central principle in rare-event modeling.

Although cumulative additive frameworks may offer advantages in terms of transparency, interpretability, and structural simplicity, important validation-related considerations remain. The present framework was not developed as a calibrated prediction model and should therefore not be interpreted as providing precise individualized probabilities of infection occurrence [11,18]. Consequently, calibration performance was not evaluated and remains an important area for future investigation.

Furthermore, the relative contribution of individual framework components may vary across institutions, patient populations, and healthcare environments. Thresholds selected on the basis of clinical reasoning and local cohort characteristics may not be directly transferable to other settings. External validation studies are therefore necessary to evaluate generalizability, transportability, and performance across diverse clinical populations before broader implementation can be considered [11,21].

A structured comparison between conventional multivariable prediction models and cumulative additive frameworks is summarized in Table 2.

The structured methodological framework illustrated in Figure 1 provides a practical representation of how risk stratification may be operationalized in clinical settings characterized by limited outcome events. By integrating baseline vulnerability assessment, exposure-related variables, cumulative score construction, and dynamic updating into a coherent workflow, the approach translates methodological principles into a reproducible implementation strategy. In this context, the emphasis shifts from coefficient optimization to structural transparency and stability, which may be more appropriate in rare-event clinical environments.

## 7. Practical Recommendations for Risk Modeling in Low-Event Clinical Cohorts

Based on the methodological considerations discussed above, several practical recommendations may be proposed for investigators developing risk stratification frameworks in clinical datasets characterized by limited outcome events.

### 7.1. Evaluate Events per Variable Before Model Construction

Prior to initiating multivariable modeling, investigators should formally assess the EPV ratio relative to the number of candidate predictors [5,7]. When EPV values fall substantially below commonly suggested stability ranges, the feasibility of coefficient-weighted regression modeling should be critically reconsidered. Explicit reporting of EPV helps contextualize model limitations and promotes methodological transparency.

### 7.2. Avoid Data-Driven Optimization in Small Datasets

In low-event cohorts, automated variable selection procedures, stepwise modeling strategies, and receiver operating characteristic-based threshold optimization may artificially inflate apparent model performance. Prespecification of clinically plausible variables and thresholds can reduce the risk of overfitting and improve structural stability [10].

### 7.3. Prioritize Structural Simplicity When Appropriate

When outcome frequency limits modeling complexity, additive cumulative frameworks may represent a defensible alternative to coefficient-weighted multivariable prediction models. Although such approaches sacrifice coefficient refinement, they enhance interpretability and reduce sensitivity to sampling variability. The trade-off between statistical precision and structural stability should be explicitly acknowledged.

### 7.4. Address Temporal Relationships Transparently

Exposure-based variables, particularly LOS and device utilization, should be interpreted with careful attention to temporal relationships. Potential reverse causality, time-dependent bias, and exposure misclassification should be considered when interpreting cumulative risk models. When time-dependent modeling is not feasible due to limited event counts, these limitations should be explicitly acknowledged and causal inference avoided.

### 7.5. Distinguish Stratification from Prediction

Researchers should clearly define whether the objective of modeling is individualized probability estimation or pragmatic risk stratification. In rare-event environments, structured categorization of patients into vulnerability strata may be more achievable and clinically useful than attempts to derive highly precise predictive algorithms. Explicit alignment between methodological strategy and intended application enhances conceptual clarity.

### 7.6. Emphasize Reproducibility over Incremental Performance Gains

Minor improvements in discrimination metrics may not justify increased model complexity in limited-event settings. Transparent and reproducible frameworks may ultimately provide greater long-term scientific value than highly optimized but structurally unstable prediction models.

Collectively, these principles emphasize the importance of methodological proportionality: the complexity of the modeling strategy should remain aligned with the informational capacity of the available data and the intended clinical application.

In the present CCU example, these considerations illustrate how cumulative additive frameworks may provide a pragmatic alternative when conventional multivariable prediction models are constrained by limited outcome events.

## 8. Conclusions

Risk modeling in rare-event clinical settings requires careful alignment between methodological ambition and the informational capacity of the available data. In CCU populations, the relatively low incidence of HAIs limits the feasibility of conventional multivariable prediction models and increases susceptibility to overfitting and coefficient instability.

Using a CCU cohort as an illustrative example, the present study demonstrates how cumulative additive frameworks may provide a pragmatic alternative when event frequency constrains model complexity. By prioritizing interpretability, transparency, and structural stability, such approaches may better align modeling strategy with data limitations and clinical usability.

Importantly, cumulative frameworks should not be interpreted as substitutes for rigorously validated prediction models. Rather, they represent a pragmatic methodological option in settings where outcome events are limited and highly parameterized models may be unreliable.

More broadly, rare-event contexts require statistical restraint, explicit acknowledgment of temporal and exposure-related biases, and careful consideration of the trade-off between predictive precision and model stability. As cardiovascular research increasingly incorporates advanced analytical approaches and artificial intelligence-assisted methodologies to improve disease characterization and clinical decision support [22], the need for transparent and clinically interpretable risk-stratification strategies remains important. Under such conditions, prioritizing structural simplicity may ultimately enhance reproducibility and practical relevance in clinical research.

The contribution of the present work lies not in the development of a novel clinical scoring system, but in the proposal of a structured methodological framework for risk stratification under low-events-per-variable conditions. Unlike established additive clinical scores, which were developed and validated for specific disease states and clinical outcomes, the present framework is intended to provide methodological guidance regarding the selection and implementation of risk-stratification strategies when outcome events are limited. Its central principle is methodological proportionality: the alignment of model complexity with the informational capacity of the available data. By emphasizing interpretability, explainability, and structural stability, the framework offers a pragmatic approach for reducing susceptibility to overfitting while maintaining clinical usability in rare-event clinical settings where conventional predictive modeling may be unstable or difficult to justify.

## Figures and Tables

**Table 1:** Distribution of HAIs across cumulative framework categories in the illustrative CCU cohort. The table summarizes the number of patients and the corresponding incidence of healthcare-associated infections (HAIs) across cumulative framework levels (0–4). Infection incidence increases progressively with higher cumulative burden, illustrating a graded pattern of increasing HAI occurrence across cumulative framework levels.

Cumulative Score	Patients (*n*)	HAI Events (*n*)	HAI Incidence (%)
0	325	0	0.0
1	242	3	1.24
2	178	5	2.81
3	100	6	6.00
4	25	2	8.00
Total	870	16	1.8

**Table 2:** Methodological comparison of multivariable regression and cumulative additive frameworks in rare-event risk modeling. The table summarizes key methodological differences between coefficient-weighted multivariable prediction models and prespecified cumulative additive frameworks when applied to datasets with limited outcome events.

Feature	Multivariable Model	Additive Cumulative Framework
Coefficient assignment	Weighted regression coefficients	Equal points per factor
Model structure	Data-driven optimization	Prespecified structure
Complexity	Higher	Lower
Overfitting risk (low EPV)	Increased	Reduced through prespecification
Interpretability	Limited (requires model coefficients)	High (transparent scoring)
Stability under small-event conditions	Sensitive to data perturbation	Structurally robust
Threshold selection	Often data-driven	Clinically prespecified

## Data Availability

The original contributions presented in this study are included in the article. Further inquiries can be directed to the corresponding author.

## Abbreviations

The following abbreviations are used in this manuscript:

AUC	Area Under the Receiver Operating Characteristic Curve
CCU	Coronary Care Unit
EPV	Events per Variable
HAI	Healthcare-Associated Infection
ICU	Intensive Care Unit
LOS	Length of Stay
LVEF	Left Ventricular Ejection Fraction
LASSO	Least Absolute Shrinkage and Selection Operator
ROC	Receiver Operating Characteristic
TRIPOD	Transparent Reporting of a Multivariable Prediction Model for Individual Prognosis or Diagnosis
